# Monkeypox (MPOX)-Related Knowledge and Vaccination Hesitancy in Non-Endemic Countries: Concise Literature Review

**DOI:** 10.3390/vaccines11020229

**Published:** 2023-01-19

**Authors:** Mohamed Lounis, Abanoub Riad

**Affiliations:** 1 Department of Agro-Veterinary Science, Faculty of Natural and Life Sciences, University of Ziane Achour, BP 3117, Road of Moudjbara, Djelfa 17000, Algeria; 2 Department of Public Health, Faculty of Medicine, Masaryk University, 625 00 Brno, Czech Republic; 3 Czech National Centre for Evidence-Based Healthcare and Knowledge Translation (Cochrane Czech Republic, Czech EBHC: JBI Centre of Excellence, Masaryk University GRADE Centre), Institute of Biostatistics and Analyses, Faculty of Medicine, Masaryk University, 625 00 Brno, Czech Republic

**Keywords:** monkeypox, knowledge, awareness, vaccine acceptance, vaccine hesitancy

## Abstract

In May 2022, the world witnessed the emergence of human monkeypox (MPOX), a new zoonotic viral disease in multiple non-endemic countries. This health threat has been associated with increased anxiety, especially after the COVID-19 catastrophe. In addition, people are exposed to an unprecedented amount of information, making them vulnerable to misinformation that may lead to embracing conspiracy theories. This literature review was conducted to evaluate the levels of MPOX-related knowledge and attitudes toward its vaccination by reviewing studies indexed in MEDLINE^®^ until 15 November 2022. A total of 16 studies conducted in non-endemic countries were included in this review, predominantly in Europe, the Middle East, and Asia. Nine studies investigated exclusively MPOX-related knowledge and awareness, and two studies were exclusively related to MPOX vaccines, while five studies dealt with both topics. The target populations were mainly healthcare professionals and the general adult population. The results revealed an unsatisfactory level of knowledge and awareness among certain groups. Regarding vaccination, the results showed that vaccine hesitancy is still common for healthcare professionals except among Chinese healthcare professionals, where the rate of vaccine acceptance was estimated at 90.1%. This review could help understand the MPOX-related knowledge and awareness and vaccine hesitancy in the first months of the emergence of the MPOX by comparing their evolution in recent studies.

## 1. Introduction

Humanity has continuously battled against infectious diseases for a long time. After the pandemic of COVID-19 and its drastic health and economic consequences, the world is again witnessing a new health threat, i.e., the re-emergence of zoonotic disease, human monkeypox disease (MPOX), in various non-endemic countries [1].

This disease is caused by the *monkeypox virus*, a double-stranded DNA virus that belongs to the *Orthopoxvirus* genus. The *Chordopoxvirinae* subfamily of the *Poxviridae* family is responsible for multiple diseases in humans and animals. *Monkeypox virus* is one of four Orthopoxvirus species pathogenic for humans with *variola*, *cowpox*, and *vaccinia* [2,3].

Despite its name, the natural reservoir of this disease is still unknown. Non-human primates (such as monkeys) are one of the main suspected reservoirs in association with other African rodents and mammals [3,4]. Further studies are required to identify the virus’ reservoir(s), its main circulation route, and its conservation in nature. A possible risk factor is eating inadequately cooked meat and other animal products of infected animals [5].

Historically, this zoonotic viral disease was first reported in 1958 from lesions of an imported macaque in a Danish laboratory, hence called monkeypox. Later, the first human case was detected in a 9-year-old child in 1970 in RDC [6]. Since then, thousands of confirmed and misdiagnosed cases in multiple outbreaks have been reported in Africa, especially in the central and western African countries (Benin, Cameroon, Central African Republic, Côte d’Ivoire, Democratic Republic of the Congo, Gabon, Ghana, Liberia, Nigeria, Republic of the Congo, South Sudan, and Sierra Leone), currently considered an endemic region (most of the cases were reported in the Republic Democratic of Congo). Consequently, two genetic strains of the *monkeypox virus* have been characterized, including the West African and the central African clade geographically separated with epidemiological and clinical differences. The number of cases and outbreaks is continually increasing in these countries, especially since the cessation of the smallpox vaccination in the 1980s [2,5,6].

Out of Africa, the first cases were reported in the USA in 2003 following the importation of infected animals from Ghana. Later, cases from different countries were also reported, including the United Kingdom (2018–2019 and 2021) and Singapore (2019) as well as in the USA in 2021 [6].

Since 6 May 2022, the world has known the re-emergence of multiple cases in different non-endemic countries with no history of travel to endemic countries. As of 10 November 2022, the number of confirmed cases had reached 79,151 [7].

The West African clade was identified as the cause for the first cases reported in non-endemic countries. Later, genome sequencing of strains from the confirmed cases in Portugal showed a close similarity with the strains isolated from exported cases from Nigeria to the United Kingdom and Singapore in 2018 and 2019 [5,8]. The virus is mainly transmitted from close contact with lesions, body fluids, respiratory droplets, and contaminated materials such as bedding [8].

Clinically, the disease is generally self-limiting with the formation of lesions, skin nodules, or disseminated rash but could be severe in some individuals, such as children, pregnant women, or immune-deficient persons [9]. The incubation period of MPOX is usually from 6 to 13 days but can range from 5 to 21 days. While the case fatality rate varied from 3.6% to 10.6% in endemic countries, very few deaths were reported in the current outbreak in non-endemic countries [6,10].

Additionally, no specific treatments or vaccines approved for MPOX are available. Some antivirals used for smallpox, e.g., tecovirimat, brincidofovir, and cidofovir, and Vaccinia Immune Globulin Intravenous (VIGIV) could be beneficial [3,11]. Additionally, vaccines against smallpox have historically shown a cross-protection against monkeypox. In this way, three vaccines that were developed against smallpox are currently used against MPOX in some countries. These vaccines, including MVA-BN (JYNNEOS), LC16, and ACAM2000, are recommended mostly for groups at high risk for exposure to MPOX [11,12].

In response to this public health threat, the World Health Organization (WHO) has released a range of recommendations to limit its spread. These recommendations are related to surveillance, case investigation and reporting, contact tracing, risk communication and community engagement, clinical management, infection prevention, and control in healthcare settings [1,5]. Later, the WHO declared the MPOX outbreak a public health emergency of international concern in July 2022 [13,14]. The rapid spread of this disease has induced anxiety among the public [15], mainly due to the lack of knowledge and the embracing of conspiracy beliefs toward emerging viral infections [16,17]. Consequently, the health authorities are again asked to communicate and convince the population to agree with preventive measures and a probable future vaccination, especially after the hard COVID-19 experience. In fact, the COVID-19 experience has shown that adhering to preventive measures is strongly associated with the level of knowledge.

Thus, the present review evaluated the levels of MPOX-related knowledge, awareness, and attitudes toward MPOX vaccines. It also aimed to highlight the associated factors of inadequate knowledge and vaccine acceptance levels.

## 2. Materials and Methods

This review was conducted following the PRISMA guidelines [18]. The papers indexed in MEDLINE ^®^ that aimed to evaluate MPOX-related knowledge, attitudes, awareness, and vaccine hesitancy/acceptance were included in this review.

The inclusion criteria were (i) English articles indexed in MEDLINE/PubMed, (ii) analytical and descriptive cross-sectional study designs, and (iii) studies aiming to evaluate MPOX-related knowledge, attitudes, awareness, and vaccine hesitancy/acceptance.

The exclusion criteria were (i) reviews, commentaries, and opinion articles and (ii) articles published in other languages than English (Figure S1).

The search strategy was applied on 15 November 2022, by combining various keywords such as: (monkeypox* knowledge*[Title/Abstract]) OR, (monkeypox* awareness *[Title/Abstract]) OR (monkeypox* attitude *[Title/Abstract]) (monkeypox *vaccine * hesitancy [Title/Abstract]) OR (monkeypox * vaccine acceptance[Title/Abstract])) OR (monkeypox * intention to vaccine * [Title/Abstract]) AND (2022:2023[pdat]) (Table S1).

At the end of the screening of titles and abstracts, data extraction was performed. Articles were classified according to the following criteria: name of the authors, country/countries in which the survey was conducted, date of survey, target population, sample size, level of knowledge, awareness and worry, tools used for evaluation of knowledge, associated factors, vaccines acceptance and its associated factors.

## 3. Results

**Figure 1:**
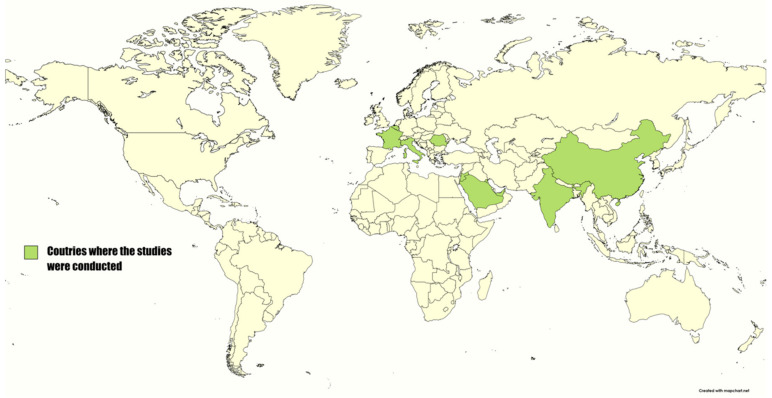
Geographic situation of the countries concerned by MPOX surveys.

The research procedure found a total of 135 published papers in the current year. At the end of the screening and selection process, 16 studies were included in this review. All the selected studies were conducted in Asia (11) or in Europe (4). They were from nine different countries, including Saudi Arabia (five studies), Jordan (two), Kuwait (one), UAE (one), China (one), India (one), Italy (two), France and Belgium (one), and Romania (one) (Figure 1).

These studies focused mainly on the awareness, knowledge, and attitudes toward MPOX vaccine. Some studies were, however, exclusively related to knowledge and awareness (nine), while two studies were exclusively related to vaccination and five studies studied the two subjects (knowledge and vaccination). Among these studies, seven studies were related to healthcare professionals, three studies were conducted among the general population, while others were conducted among certain categories including students (one), medical students (two), and adults (one). The studies were conducted between May and August 2022 using in general an online questionnaire with a sample size varying from 314 to 1546 individuals (Table 1).

### 3.1. MPOX-Related Knowledge and Anxiety

The results of the different studies showed that different levels of awareness were obtained. In fact, according to the studies of Kaur et al. [27] and Ricco et al. [30], 24.8% of dental professionals and 27% of healthcare professionals, respectively, never heard about monkeypox disease before this pandemic. Additionally, the level of awareness about the current epidemic could be low among healthcare professionals (45.05%) [16], dental professionals (39.5%), and health students (50.24%) [16]. This level is lower among the general population where 26.7% of Italian adults heard about the current outbreak of MPOX as reported by Gallé et al. [24]. Another study was based, however, on self-reported awareness where the participants declared that they have a low (4.1%), moderate (36.8%), or high (23.24%) level of awareness [19].

The level of knowledge was estimated in the different studies using different scales. These scores or the level of knowledge were conducted by estimating the level of correct responses to some items related to MPOX ranging from 9 to 27 items (Table 2).

These items are related to the etiology, epidemiology and transmission, clinical signs, treatment, and preventive measures. The authors generally qualify a high level of knowledge at a level higher than the median level of knowledge of the studied sample.

Overall, a poor to medium level of knowledge was obtained in almost all studies. The percentage of the population with a high score varied from 22.8% [22] to 56% [15]. The lowest score was obtained among university students, while the highest score was obtained from the general population.

Multiple factors were associated with high levels of knowledge. Age and educational level were the most cited factors [16,21,22,24,26,27,31]. Other factors were also cited and were mainly related to the professional position and profile, the source of information, and conspiracy beliefs [31].

Regarding sex, the results of two studies were opposed. Sallam et al. [16] reported that males were more informed, while Jairoun et al. [26] showed that females had the highest level of knowledge. The latter category (females), as well as individuals who were not infected with COVID-19 and medical students, are more worried about human monkeypox as reported by Aljamaan et al. [19].

In this way, the level of worry varied among different studies. This level varied from 26.37% of the total population [29] and 28.5% among French and Belgian healthcare professionals [23] to 60.4% among the general population in Saudi Arabia [15]. Moreover, 37.5% of the studied Saudi healthcare workers were more concerned about MPOX than about COVID-19 [19].

### 3.2. MPOX Vaccine Hesitancy

The results obtained from the different studies showed that the lowest vaccination acceptance rate was obtained in the general population in Romania 29% [29], while the highest rate was obtained among healthcare professionals in China (90.1%) [25] (Table 3).

The rate of acceptance/willingness in the general population varied from 29% [29] to 50.6% [15], while it varied from 55.4% [23] to 90.1% in healthcare professionals [25]. Additionally, 55.4% of French and Belgian healthcare professionals agree to be vaccinated. The rate of acceptance reaches 79.1% in the case of the spread of MPOX within the general population [23].

Regarding associated factors with MPOX vaccine acceptance, the results showed that COVID-19 infection and worry, professional place occupation, age, educational level, Influenza vaccination, and some beliefs are all predictor factors of acceptance/willingness to vaccinate.

## 4. Discussion

While the world has not yet recovered from the COVID-19 pandemic, the current MPOX outbreak in some non-endemic countries provoked real concern. Known as an endemic disease in central–western African countries since the 1970s, the disease has been reported out of Africa since 2003 in multiple countries with a link to travel to endemic countries. However, the re-emergence of the disease in non-endemic countries in recent years has attracted more concern for multiple reasons: first, multiple sporadic cases were reported simultaneously in different countries with no link to travel to endemic countries; second, the rapid spreading of this disease, the zoonotic character, and the lessons learned from the COVID-19 were concerning.

Thus, the current review aimed to report the level of knowledge and awareness in non-endemic countries and the attitudes toward MPOX vaccination.

The findings of this review showed a moderate level of awareness about MPOX. If the fact that 24.8% and 27% of dental professionals [27] and healthcare professionals [30], respectively, never heard about monkeypox disease before the current outbreak is explicable due to the disease being typically reported in endemic countries, the low level of awareness about the current outbreak reported among healthcare professionals and medical students is surprising. These results could be related to the fact that these studies were conducted during the first months of the emergence of the diseases, and no cases were reported in the countries where they were conducted [16,27,31]. The level of awareness could be as low as 26.7% among adults [24].

Regarding the knowledge about MPOX in the different studies, even though multiple scales were used to assess the level of knowledge, the results generally showed a poor to moderate level even among healthcare professionals and university students [21,31]. This low level of knowledge could be explained by the fact that the population in non-endemic countries is in the discovering stage of MPOX.

The low level of knowledge obtained among healthcare professionals in some studies is, however, alarming. This category is considered a key group in the fight against and prevention of the spread of health threats, especially following the emergence of new infectious diseases [16,32]. This category also represents the main source of information about health threats for the general population and thus plays an important role in raising knowledge, and it is the main partner in any awareness campaigns. Of note, the same observation was reported in Indonesia in some studies conducted in 2020 [33,34].

Some of the selected studies of this review have, however, shown that being a healthcare worker is associated with a high level of knowledge in the general population [21] and physicians are more informed [20].

In addition, other factors were associated with a high level of knowledge. Even though the categories were not standardized in the different studies, older individuals and those with higher educational levels were shown to be more informed about MPOX than their counterparts [16,20,22,24,26,27,31]. Other factors were also cited, including the source of information and conspiracy beliefs. In fact, conspiracy theories and beliefs are the main sources of acquiring misinformation and thus reduce the level of knowledge and awareness [16].

For the effect of sex on the level of knowledge, most of the studies failed to find a statistical relationship between sex and MPOX knowledge, while the results of Sallam et al. [16] were in favor of males and those of Jairoun et al. [26] were in favor of females. Likewise, females were more worried about MPOX than males. These findings may be due to the fact that females were reported to believe more in rumors and conspiracy theories than males [31]. Medical students and individuals not infected with COVID-19 were also more worried about human monkeypox [19].

Regarding the attitude toward vaccination, a high level of acceptance among healthcare professionals in China (90.1%) is apparent. These results may be explained by the experience of China in the fight against emerging diseases, especially after the two experiences of SARS (Severe Acute Respiratory Syndrome) and COVID-19.

Moreover, healthcare professionals were more likely to accept MPOX vaccination than the general population. These results make sense knowing that healthcare professionals are on the frontline in fighting any health threat, and thus, they are exposed to the risk of contamination. As a consequence, they are prioritized in any vaccination strategy, as was the case with COVID-19. These results are in accordance with the results of previous studies conducted in Indonesia before the COVID-19 pandemic where the rate of acceptance could reach 96% [35,36].

The second important result is the high rate of hesitancy among the general population, especially in Romania where only 29% had a favorable attitude toward vaccination [26]. These low rates of acceptance/willingness agree with the rates obtained for COVID-19 vaccines, especially in the first months of their approvals. National and international health authorities should make more of an effort to sensitize the population to the benefits of vaccines in the struggle against infectious diseases, and the latest example is the COVID-19 pandemic. Of note, the phenomenon of vaccine hesitancy is classified as one of the top 10 public health threats by the WHO [37].

The selected studies reported some factors that were in favor of vaccination which include COVID-19 infection and worry, professional place occupation, age, educational level, Influenza vaccination, and some beliefs, which could help in the strategy of fighting the phenomenon of vaccine hesitancy by raising awareness about the importance of vaccination targeting the hesitant categories.

This review presents some strengths and limitations. Regarding the strengths, to our knowledge, this is the first review of its nature to deal with monkeypox knowledge and attitudes toward vaccination against it in non-endemic countries. In addition, the review was conducted using PRISMA guidelines. Thus, the findings of this review could be used as a baseline in estimating knowledge and vaccine acceptance in the first months of the MPOX outbreak.

The limitations of this review are mainly due to including only studies available in MEDLINE. It is, however, conducted in one of the most crucial research engines on medical and biological studies, allowing to include only indexed studies and making this review concise. In addition, most of these studies were conducted in the initial stage of the MPOX outbreak; thus, the level of knowledge and awareness could increase over time. Another limitation is the evaluation of the level of knowledge where different scales were used, making the comparison between the different reasons inadequate. Finally, the acceptance rate was evaluated in the absence of a specific vaccine against MPOX, which could change according to the evolution of the situation and the probable approval and introduction of a new MPOX vaccine. Moreover, a quantitative synthesis was not planned to be performed in this review. Therefore, a critical appraisal of the included studies was not performed.

## 5. Conclusions

The ongoing MPOX outbreak has attracted worldwide concern. After the COVID-19 experience, people are becoming more aware of emerging and re-emerging diseases. However, the large amount of available information and its accessibility expose the population to false and non-scientific ideas and conspiracy theories. The current review has shown that the level of knowledge and awareness is unsatisfactory even among healthcare professionals. Additionally, except for one study in China showing high acceptance among healthcare professionals, vaccine hesitancy is still common among healthcare professionals and the general population in other countries. Thus, increasing the level of knowledge and fighting the phenomenon of vaccine hesitancy by targeting the categories with the lowest levels of knowledge and vaccine acceptance could help to fight against MPOX specifically and other future infectious diseases in general. This review could be a baseline for human monkeypox knowledge, awareness, and vaccine acceptance for future studies. Thus, future reviews, rigorously following PRISMA guidelines and with updated data, could be very helpful to evaluate the level of knowledge and the vaccine acceptance evolution. Additionally, highlighting the role of other sociodemographics, such as religiosity, the endemicity of infectious diseases, and confidence in governments in future studies is necessary for the understanding of the phenomenon of vaccine hesitancy.

## Figures and Tables

**Table 1:** General Characteristics of the Included Studies.

First Author	Country	Timeframe of Data Collection	Target Population	Sample Size	Statistical Analysis/Design	Objectives
Aljamaan et al. [19]	Saudi Arabia	27 May–10 June 2022	Healthcare professionals	1130	multivariate logistic binary	Knowledge and Vaccine Attitudes
Alsanafi et al. [20]	Kuwait	July–August 2022	Healthcare professionals	896	multivariate logistic binary	Knowledge and Confidence in Diagnosis and Management
Alshahrani et al. [21]	Saudi Arabia	25 May–15 July 2022	General population	480	Chi-Square test	Knowledge
Alshahrani et al. [22]	Saudi Arabia	May–July 2022	Medical students	314	Pearson’s Chi-square test	Knowledge and Perceptions
Gagneux-Brunon et al. [23]	France and Belgium	15 June–8 August 2022	Healthcare professionals	690	multivariate logistic binary	Knowledge, Anxiety and Vaccine Attitudes
Gallè et al. [24]	Italy	July–August 2022	General population	1352	multivariate logistic regression	Anxiety and Vaccine Attitudes
Hong et al. [25]	China	30 May–1 August 2022	Healthcare professionals	1032	multivariate logistic regression analysis	Vaccine Attitudes
Jairoun et al. [26]	UAE	15 May–28 May 2022	University students	558	multivariate logistic regression analysis to	Knowledge
Kaur et al. [27]	India	June 2022	Dental professionals	410	ANOVA and Chi-square test	Knowledge
Meo et al. [28]	Saudi Arabia	15 May–15 July 2022	General population	1020	t-tests, ANOVA, and chi-squared tests	Knowledge and Perceptions
Peptan et al. [29]	Romania	1 July–31 July 2022	General population	820	Kendell and Spearman tests (correlation)	Vaccine Attitudes
Riccò et al. [30]	Italy	May 2022	General physicians	163	multivariable logistic binary	Knowledge and Vaccine Attitudes
Sallam et al. [31]	Jordan	May 2022	Healthcare students	615	multinomial regression analysis	Knowledge and Conspiracy Beliefs
Sallam et al. [16]	Jordan	May–July 2022	Healthcare professionals	606	Univariate regression analysis	Knowledge and Confidence in Diagnosis and Management
Temsah et al. [15]	Saudi Arabia	27 May–5 June 2022	General population	1546	Multivariate Binary Logistic Regression	Knowledge, Anxiety and Vaccine Attitudes

**Table 2:** MPOX-related Knowledge According to the Included Studies.

First Author	Instrument	Knowledge Level	Independent Variables	Anxiety
Aljamaan et al. [19]	N/A	N/A	The high level of knowledge was associated with being female, those working in medical field, and those who were not affected by COVID-19	37.5% were more concerned about MPOX than COVID-19
Alsanafi et al. [20]	10 items	Unsatisfactory	Physicians have the highest levels of knowledge compared with the other categories	N/A
Alshahrani et al. [21]	23 items	High: 48%	The highest level of knowledge was associated with older individuals, married, those living in urban areas, postgraduates, employed, healthcare professionals, those with high income, and smokers	N/A
Alshahrani et al. [22]	20 items	High: 28%	The highest level of knowledge was associated with individuals aged more than 21 years and those who had contracted COVID-19	N/A
Gallè et al. [24]	*N/A*	High: 48.15%	The highest level of knowledge was associated with individuals aged more than 53 years, those working/studying in non-healthcare settings, singles, and those having mass media as the main source of information	N/A
Jairoun et al. [26]	27 items	High: 22.8%	The highest level was associated with older students, females, medical students, those having a history of human chickenpox infection, and those receiving information about MPOX in their education	N/A
Kaur et al. [27]	12 items	High: 28%	The highest level was associated with postgraduates and academicians/teachers	N/A
Meo et al. [28]	13 items	Satisfactory	N/A	40.4% were afraid of MPOX
Peptan et al. [29]	N/A	N/A	N/A	26.4% expressed their fear of becoming infected
Riccò et al. [30]	24 items	Unsatisfactory	N/A	30.1% perceived MPOX would become a likely occurrence during daily activities; 32.5% perceived that it could potentially affect them
Sallam et al. [31]	11 items	Unsatisfactory	The highest level was associated with students aged more than 21 years	N/A
Sallam et al. [16]	11 items	Unsatisfactory	The highest level was associated with males and postgraduates	N/A
Temsah et al. [15]	9 items	High: 56%	N/A	60.4% were worried about the progression of the disease into a global pandemic

**Table 3:** MPOX-related Vaccine Attitudes According to the Included Studies.

First Author	Vaccine Acceptance Level	Independent Variables
Aljamaan et al. [19]	69.8% *	The high level of vaccines recommendation was associated with individuals who contracted COVID-19
Gagneux-Brunon et al. [23]	55.4% (in the period of the study) 79.1% In the case of spread within the general population,	The high level of acceptance was associated with physicians or pharmacists
Gallè et al. [24]	45.8%	N/A
Hong et al. [25]	90.1%	The high level of acceptance was associated with individuals aged 30–40 years, those working in secondary hospitals, those who consider vaccination necessary, those willing to pay for the vaccine, those considering mandatory vaccination necessary, and those recommending vaccination to their family
Meo et al. [28]	43.7% (those recommending vaccination)	The high level of vaccination recommendation was associated with postgraduates (PhD/Fellowship)
Peptan et al. [29]	29.3%	N/A
Riccò et al. [30]	58.6% (somehow favorable)	The high level of vaccine acceptance was associated with individuals previously vaccinated against seasonal influenza and those being favorable to receive variola vaccine
Temsah et al. [15]	50.6% (agree with vaccination)	The high level of acceptance was associated with individuals less than 45 years old, those without a university degree, those with moderate to high levels of self and family commitment to infection control precautionary measures, those who expressed self and family worry about MPOX infection, those who searched more for information about MPOX, and those considering the ministry of health as a source of information

* The participating healthcare workers were asked if they should be prioritized for the MPOX vaccine.

## Data Availability

The data supporting this study’s findings are available from the corresponding author (M.L.) upon reasonable request.

## Supplementary Materials

The following supporting information can be downloaded at: https://www.mdpi.com/article/10.3390/vaccines11020229/s1, Figure S1: Flow diagram of the study selection process; Table S1: Search strategies.

## References

[1] World Health Organization (WHO) Multi-Country Monkeypox Outbreak in Non-Endemic Countries. https://www.who.int/emergencies/disease-outbreak-news/item/2022-DON385.

[2] Sklenovská N., van Ranst M. (2018). Emergence of Monkeypox as the Most Important Orthopoxvirus Infection in Humans. Front. Public Health.

[3] Centers for Disease Control and Prevention (CDC) Monkeypox | Poxvirus | CDC. https://www.cdc.gov/poxvirus/monkeypox/vaccines.html.

[4] Bonilla-Aldana D.K., Rodriguez-Morales A.J. (2022). Is Monkeypox Another Re-emerging Viral Zoonosis with Many Animal Hosts yet to Be Defined?. Vet. Q..

[5] World Health Organization (WHO) Multi-Country Monkeypox Outbreak in Non-Endemic Countries: Update. https://www.who.int/emergencies/disease-outbreak-news/item/2022-DON388.

[6] Bunge E.M., Hoet B., Chen L., Lienert F., Weidenthaler H., Baer L.R., Steffen R. (2022). The Changing Epidemiology of Human Monkeypox—A Potential Threat? A Systematic Review. PLoS Negl. Trop. Dis..

[7] Mathieu E., Spooner F., Dattani S., Ritchie H., Roser M. (2022). Mpox (Monkeypox). Our World Data. https://ourworldindata.org/monkeypox.

[8] Antunes F., Virgolino A. (2022). Monkeypox Mysteries of the New Outbreak in Non-Endemic Areas. Int. J. Environ. Res. Public Health.

[9] Riad A., Attia S. (2023). Monkeypox-Related Oral Manifestations and Implications: Should Dentists Keep an Eye Out?. J. Med. Virol..

[10] Farahat R.A., Abdelaal A., Shah J., Ghozy S., Sah R., Bonilla-Aldana D.K., Rodriguez-Morales A.J., McHugh T.D., Leblebicioglu H. (2022). Monkeypox Outbreaks during COVID-19 Pandemic: Are We Looking at an Independent Phenomenon or an Overlapping Pandemic?. Ann. Clin. Microbiol. Antimicrob..

[11] Rizk J.G., Lippi G., Henry B.M., Forthal D.N., Rizk Y. (2022). Prevention and Treatment of Monkeypox. Drugs.

[12] Overton E.T., Lawrence S., Stapleton J., Weidenthaler H., Schmidt D., Nopora K., Meyer T., Maclennan J., Koenen B., Silbernagl G. (2020). MVA-BN as Monkeypox Vaccine for Healthy and Immunocompromised. Int. J. Infect. Dis..

[13] World Health Organization (WHO) WHO Director-General’s Statement at the Press Conference Following IHR Emergency Committee Regarding the Multi-Country Outbreak of Monkeypox—23 July 2022. https://www.who.int/director-general/speeches/detail/who-director-general-s-statement-on-the-press-conference-following-IHR-emergency-committee-regarding-the-multi--country-outbreak-of-monkeypox--23-july-2022.

[14] Centers for Disease Control and Prevention (CDC) 2022 Monkeypox Outbreak Global Map. https://www.cdc.gov/poxvirus/monkeypox/response/2022/world-map.html.

[15] Temsah M.H., Aljamaan F., Alenezi S., Alhasan K., Saddik B., Al-Barag A., Alhaboob A., Bahabri N., Alshahrani F., Alrabiaah A. (2022). Monkeypox Caused Less Worry than COVID-19 among the General Population during the First Month of the WHO Monkeypox Alert: Experience from Saudi Arabia. Travel Med. Infect. Dis..

[16] Sallam M., Al-Mahzoum K., Al-Tammemi A.B., Alkurtas M., Mirzaei F., Kareem N., Al-Naimat H., Jardaneh L., Al-Majali L., AlHadidi A. (2022). Assessing Healthcare Workers’ Knowledge and Their Confidence in the Diagnosis and Management of Human Monkeypox: A Cross-Sectional Study in a Middle Eastern Country. Healthcare.

[17] Riad A., Drobov A., Rozmarinová J., Drapáčová P., Klugarová J., Dušek L., Pokorná A., Klugar M. (2022). Monkeypox Knowledge and Vaccine Hesitancy of Czech Healthcare Workers: A Health Belief Model (HBM)-Based Study. Vaccines.

[18] Page M.J., McKenzie J.E., Bossuyt P.M., Boutron I., Hoffmann T.C., Mulrow C.D., Shamseer L., Tetzlaff J.M., Akl E.A., Brennan S.E. (2021). The PRISMA 2020 Statement: An Updated Guideline for Reporting Systematic Reviews. BMJ.

[19] Aljamaan F., Alenezi S., Alhasan K., Saddik B., Alhaboob A., Altawil E.S., Alshahrani F., Alrabiaah A., Alaraj A., Alkriadees K. (2022). Healthcare Workers’ Worries and Monkeypox Vaccine Advocacy during the First Month of the WHO Monkeypox Alert: Cross-Sectional Survey in Saudi Arabia. Vaccines.

[20] Alsanafi M., Al-Mahzoum K., Sallam M. (2022). Monkeypox Knowledge and Confidence in Diagnosis and Management with Evaluation of Emerging Virus Infection Conspiracies among Health Professionals in Kuwait. Pathogens.

[21] Alshahrani N.Z., Alzahrani F., Alarifi A.M., Algethami M.R., Alhumam M.N., Ayied H.A.M., Awan A.Z., Almutairi A.F., Bamakhrama S.A., Almushari B.S. (2022). Assessment of Knowledge of Monkeypox Viral Infection among the General Population in Saudi Arabia. Pathogens.

[22] Alshahrani N.Z., Mitra S., Alkuwaiti A.A., Alhumam M.N., Altmimi S.M.B., Alamri M.H.M., Albalawi Z.A.S., Almorgi M.W., Alharbi H.K.D., Alshahrani S.M. (2022). Medical Students’ Perception Regarding the Re-Emerging Monkeypox Virus: An Institution-Based Cross-Sectional Study From Saudi Arabia. Cureus.

[23] Gagneux-Brunon A., Dauby N., Launay O., Botelho-Nevers E. (2022). Attitudes towards Monkeypox Vaccination among Healthcare Workers in France and Belgium: An Element of Complacency?. J. Hosp. Infect..

[24] Gallè F., Bianco L., da Molin G., Mancini R., Sciacchitano S., Ferracuti S., Liguori G., Orsi G.B., Napoli C. (2022). “Monkeypox: What Do You Know about That?” Italian Adults’ Awareness of a New Epidemic. Pathogens.

[25] Hong J., Pan B., Jiang H.-J., Zhang Q.-M., Xu X.-W., Jiang H., Ye J., Cui Y., Yan X.-J., Zhai X.-F. (2023). The Willingness of Chinese Healthcare Workers to Receive Monkeypox Vaccine and Its Independent Predictors: A Cross-Sectional Survey. J. Med. Virol..

[26] Jairoun A.A., Al-Hemyari S.S., Abdulla N.M., El-Dahiyat F., Shahwan M., Hassan N., Jairoun O., Alyousef N.G., Sharif S., Jaber A.A.S. (2022). Awareness and Preparedness of Human Monkeypox Outbreak among University Student: Time to Worry or One to Ignore?. J. Infect. Public Health.

[27] Kaur A., Goel R., Singh R., Bhardwaj A., Kumari R., Gambhir R.S. (2022). Identifying Monkeypox: Do Dental Professionals Have Adequate Knowledge and Awareness?. Rocz Panstw Zakl Hig.

[28] Meo S.A., Al-Khlaiwi T., Aljofan Z.F., Alanazi A.I., Meo A.S. (2022). Public Perceptions of the Emerging Human Monkeypox Disease and Vaccination in Riyadh, Saudi Arabia: A Cross-Sectional Study. Vaccines.

[29] Peptan C., Băleanu V.D., Mărcău F.C. (2022). Study on the Vaccination of the Population of Romania against Monkeypox in Terms of Medical Security. Vaccines.

[30] Riccò M., Ferraro P., Camisa V., Satta E., Zaniboni A., Ranzieri S., Baldassarre A., Zaffina S., Marchesi F. (2022). When a Neglected Tropical Disease Goes Global: Knowledge, Attitudes and Practices of Italian Physicians towards Monkeypox, Preliminary Results. Trop. Med. Infect. Dis..

[31] Sallam M., Al-Mahzoum K., Dardas L.A., Al-Tammemi A.B., Al-Majali L., Al-Naimat H., Jardaneh L., AlHadidi F., Al-Salahat K., Al-Ajlouni E. (2022). Knowledge of Human Monkeypox and Its Relation to Conspiracy Beliefs among Students in Jordanian Health Schools: Filling the Knowledge Gap on Emerging Zoonotic Viruses. Medicina.

[32] Wilkason C., Lee C., Sauer L.M., Nuzzo J., McClelland A. (2020). Assessing and Reducing Risk to Healthcare Workers in Outbreaks. Health Secur..

[33] Harapan H., Setiawan A.M., Yufika A., Anwar S., Wahyuni S., Asrizal F.W., Sufri M.R., Putra R.P., Wijayanti N.P., Salwiyadi S. (2020). Confidence in Managing Human Monkeypox Cases in Asia: A Cross-Sectional Survey among General Practitioners in Indonesia. Acta Trop..

[34] Harapan H., Setiawan A.M., Yufika A., Anwar S., Wahyuni S., Asrizal F.W., Sufri M.R., Putra R.P., Wijayanti N.P., Salwiyadi S. (2020). Knowledge of Human Monkeypox Viral Infection among General Practitioners: A Cross-Sectional Study in Indonesia. Pathog. Glob. Health.

[35] Harapan H., Wagner A.L., Yufika A., Setiawan A.M., Anwar S., Wahyuni S., Asrizal F.W., Sufri M.R., Putra R.P., Wijayanti N.P. (2020). Acceptance and Willingness to Pay for a Hypothetical Vaccine against Monkeypox Viral Infection among Frontline Physicians: A Cross-Sectional Study in Indonesia. Vaccine.

[36] Harapan H., Setiawan A.M., Yufika A., Anwar S., Wahyuni S., Asrizal F.W., Sufri M.R., Putra R.P., Wijayanti N.P., Salwiyadi S. (2020). Physicians’ Willingness to Be Vaccinated with a Smallpox Vaccine to Prevent Monkeypox Viral Infection: A Cross-Sectional Study in Indonesia. Clin. Epidemiol. Glob. Health.

[37] World Health Organization (WHO) Ten Threats to Global Health in 2019. https://www.who.int/news-room/spotlight/ten-threats-to-global-health-in-2019.

