# Supplementary material for: Monkeypox (MPOX)-Related Knowledge and Vaccination Hesitancy in Non-Endemic Countries: Concise Literature Review
Source: Vaccines (Basel). 2023 Jan 19;11(2):229. doi: 10.3390/vaccines11020229 (PMC9958664; doi:10.3390/vaccines11020229)
Supplement: Supplementary file 1 [file vaccines-11-00229-s001.zip › vaccines-2130720-supplementary.pdf]

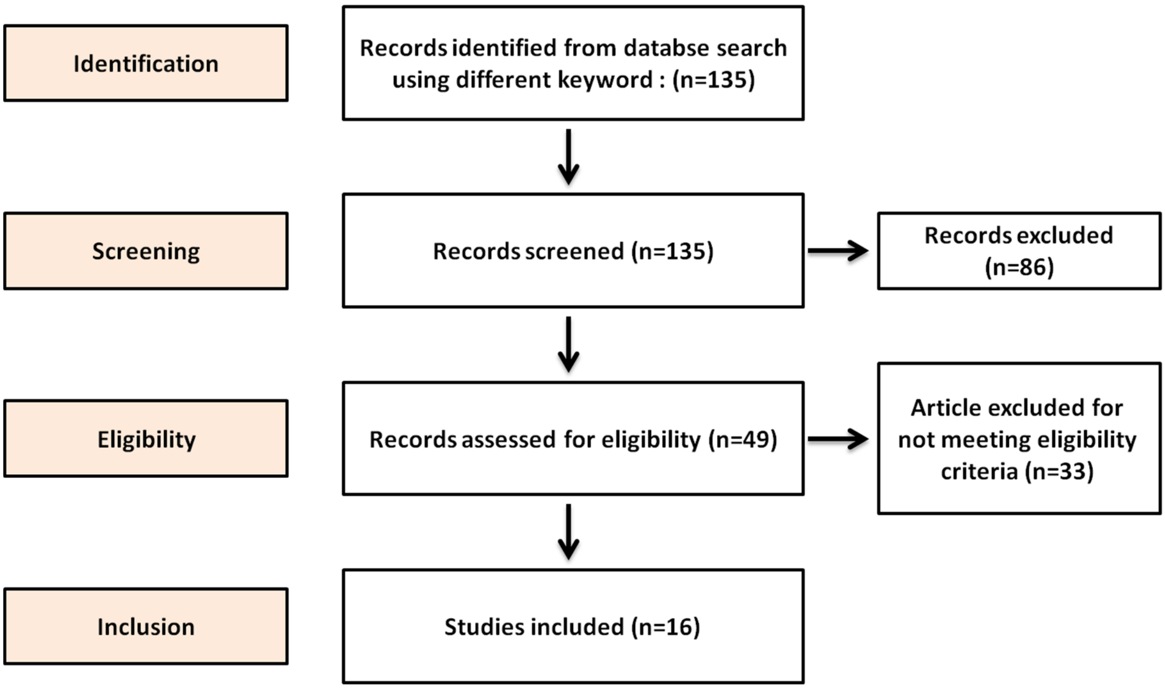


**Figure S1.** Flow diagram of the study selection process

**Table S1.** Search strategies

| Search | **Formula** | Number of studies |
|---|---|---|
| 1 | monkeypox* knowledge*[Title/Abstract] | 8 |
| 2 | monkeypox* awareness *[Title/Abstract] | 13 |
| 3 | monkeypox* attitude *[Title/Abstract | 3 |
| 4 | monkeypox *vaccine * hesitancy [Title/Abstract] | 48 |
| 5 | monkeypox * vaccine acceptance[Title/Abstract] | 15 |
| 6 | monkeypox * intention to vaccine * [Title/Abstract] | 48 |
